# Unveiling the potential of novel yeast protein extracts in white wines clarification and stabilization

**DOI:** 10.3389/fchem.2015.00020

**Published:** 2015-03-18

**Authors:** Joana P. Fernandes, Rodrigo Neto, Filipe Centeno, Maria De Fátima Teixeira, Ana Catarina Gomes

**Affiliations:** ^1^Genomics Unit, BiocantCantanhede, Portugal; ^2^PROENOL - Indústria Biotecnológica, Lda.Canelas, Portugal

**Keywords:** browning potential, clarification, fining agents, oxidation, protein content, white wine, yeast protein extracts

## Abstract

Fining agents derived from animal and mineral sources are widely used to clarify and stabilize white wines. Nevertheless, health and environmental problems are being raised, concerning the allergenic and environmental impact of some of those fining products. In this study, our aim is to validate the potential of yeast protein extracts, obtained from an alternative and safe source, naturally present in wine: oenological yeasts. Three untreated white wines were used in this work in order to evaluate the impact of these novel yeast protein extracts (YPE) in terms of the wine clarification and stabilization improvement. Two separated fining trials were thus conducted at laboratory scale and the yeast alternatives were compared with reference fining agents, obtained from mineral, animal and vegetable origins. Our results indicate that YPE were capable to promote (i) brilliance/color improvement, (ii) turbidity reduction (76–89% comparing with the untreated wines), and (iii) production of compact and homogeneous lees (44% smaller volume than obtained with bentonite). Additionally, after submitting wines to natural and forced oxidations, YPE treatments revealed (iv) different forms of colloidal stabilization, by presenting comparable or superior effects when particularly compared to casein. Altogether, this study reveals that YPE represent a promising alternative for white wine fining, since they are resultant from a natural and more sustainable origin, at present not regarded as potential allergenic according to Regulation (EC) No. 1169/2011.

## Introduction

The wine industry is one of the most competitive sectors all over the world. Accordingly, innovative oenological products and techniques constantly need to be optimized in order to produce high quality wines that are able to fulfill the demanding consumers. Particularly, white wines should present high levels of brilliance, a non-oxidized and pleasant final color and a balanced organoleptic profile (Bonilla et al., 2001; Oliveira et al., 2011; Cosme et al., 2012). In order to promote efficient clarification and stabilization processes, fining is still one of the most traditionally used techniques and therefore, a wide range of diverse fining agent formulations have been developed during the last decades (Braga et al., 2007; Cosme et al., 2007; Marangon et al., 2012; Lucchetta et al., 2013). Currently, fining products might be composed by animal, mineral and vegetable particles, or macromolecules (Iturmendi et al., 2010; Gambuti et al., 2012) or even by mixed formulations that combine a miscellaneous of distinct compounds, trying to simultaneously promote parallel effects on wines.

Animal fining agents as casein and caseinates are well-characterized to efficiently interact with problematic oxidizable compounds, due to their affinity to specific flavanol monomers and low molecular weight procyanidins (Cosme et al., 2008). By removing these undesired compounds that remain in the wine matter after fermentation, they are able to prevent the browning phenomenon after bottling. Despite their efficiency, some animal origin products as casein were shown to cause allergenic concerns on the consumers' health (Patzl-Fischerleitner and Eder, 2012; Schumann et al., 2013; Deckwart et al., 2014) and therefore, recent European legislations have already been adopted stating that if egg or milk derived proteins are detected on final wines, they must be declared on the respective bottle label [regulation (EU) No 579/2012]. On the other side, bentonite (montmorillonite mineral) is a non-protein fining agent that reduces the haze phenomenon in white through electrostatic adsorption to unstable proteins positively charged at wine pH (Blade and Boulton, 1988; Achaerandio et al., 2001; Sauvage et al., 2010; Pocock et al., 2011). These specific grape proteins remain in the wine until the end of fermentation, due to their highly resistance to proteases and to the low wine pH, but can aggregate into light-dispersing particles under elevated temperatures during wine storage or transportation (Waters et al., 1992; Pocock et al., 2000; Le Bourse et al., 2011; Marangon et al., 2011). Although it is one of the most used fining agents, bentonite is already described to reduce essential aromatic compounds and consequently to compromise the wine final quality (Armada and Falque, 2007). Other problems involved with bentonite fining include long settling times, the associated manual handling requirements and the environmental costs for disposal of its waste (Hsu and Heatherbell, 1987; Marangon et al., 2012; Lucchetta et al., 2013).

In order to overcome the health and environmental concerns related with the use of the animal and mineral origin products, biological, and more environmental-friendly alternatives should be further investigated (Marchal et al., 2002; Iturmendi et al., 2010; Patzl-Fischerleitner and Eder, 2012; Schumann et al., 2013). In fact, the wine sector has already made some efforts trying to find an efficient alternative to animal and mineral origin products. The application of particular vegetable fining alternatives extracted from wheat, pea, and potato origin was already authorized by the International Organization of Vine and Wine (OIV) [Resolution OIV-OENO 495-2013]. More recently, proteins extracted from potato and grape seed actually showed some promising results in reducing harshness and astringency in red wines (Gambuti et al., 2012; Vincenzi et al., 2013). Regarding white wines, no efficient fining alternatives were still found to simultaneous clarification and stabilization, without necessity to add enological adjuvants during the fining process (Marchal et al., 2002; Cosme et al., 2012; Gambuti et al., 2012).

Alternatively, former studies explored novel fining treatments based in the application of yeast derivatives (e.g., cell wall components or yeast proteins), which have resulted in wine improvements such as a turbidity decrease, an astringency reduction and a stabilization potential (Dupin et al., 2000a; Bonilla et al., 2001; Caridi, 2006; Iturmendi et al., 2010). In fact, yeast plays a primary role in the winemaking process and thus, after fermentation, its autolysis naturally leads to a release of diverse cell compounds as enzymes, mannoproteins, fatty acids, nucleotides, peptides, and free amino acids (Zhang et al., 2011). Nevertheless, *Saccharomyces cerevisiae* extracts are known to contain some particular proteins that are potentially involved in allergic responses (Lindberg et al., 1992; Kortekangas-Savolainen et al., 1993; Nermes et al., 1995; Savolainen et al., 1998; Nittner-Marszalska et al., 2001), namely the glycolytic enzymes, Enolase 1 (ENO1), and Enolase 2 (ENO2) that are involved in carbohydrate metabolism and therefore, intrinsic to the wine fermentation process (Varela et al., 2005; Kornblatt et al., 2013), contrary to all the exogenous products that are commonly applied and already described to raise both health and environmental problems.

In this work, our aim is to evaluate the potential of two alternative yeast protein extracts (YPE), derived from a wine native source—oenological yeasts—as potential alternatives to problematic reference fining agents commonly used in white wines. For that purpose, the impact of the YPE was measured in terms of different oenological parameters as turbidity, chromatic characteristics, lees volume, protein content, browning/oxidation prevention, and curative potential after wine oxidation. The production of these two previously selected YPE is currently being implemented at industrial scale to efficiently confirm their potential as innovative and natural fining agents for application in white wines, already authorized by the European Union [regulation (EU) No 144/2013].

## Materials and methods

### Wines and fining agents

Three young white wines (2013) were intentionally selected from three distinct Portuguese regions—Dão, Lisbon and Algarve—in order to cover different wine patterns and characteristics.

Those wines were received 1 week after wine alcoholic fermentation was completed and therefore, they were still turbid and unstable. Until used, they were stored at 14 ± 2°C and the sulfur dioxide was rectified to avoid their premature evolution. Four fining products were used as reference for the fining experiments and prepared according to the supplier recommendations. These enological products were composed by different elements, respectively: Bentonite, Casein, Polyvinylpolypyrrolidone – PVPP, and Vegetable protein derived from hydrolyzed wheat gluten. Wines and fining agents were kindly provided by Proenol (Canelas, Portugal).

### Yeast protein extracts (YPE)

According to our preliminary fining trials, two specific yeast strains were selected from a diverse collection of oenological yeasts, all isolated from spontaneous wine fermentations. The corresponding protein extracts, namely BCV1 and BCV5, were produced through a confidential methodology developed at laboratory scale. Those yeast protein extracts were obtained in optimized liquid solutions with the final protein concentration of 50 g/L.

### Fining experiments

#### After alcoholic fermentation

After receiving the three untreated wines, the reference fining agents (x4) and the YPE (x2) were simultaneously tested. Fining assays were conducted in triplicates, during 48 h, using glass bottles of 275 ml of total volume. Just before the fining trials, each bottle was filled in with 250 ml of wine and the experiments were conducted at a controlled temperature of 22 ± 2°C. For the application of the reference fining agents, the dosages (minimum and maximum) were defined according to the supplier recommendations. In the case of the two YPE, the fining dosages were determined according to the previously fining experiments performed at laboratory scale. The tested dosages are presented in Table 1. Fining substances were left to flocculate and sediment to the bottom of the bottles during 48 h. Clarified wine samples (supernatants) were then gently pipetted and filtered through a qualitative paper filter, pore size 11 μm (Whatman™ Grade 1) to new, clean bottles. Wine samples were posteriorly analyzed by the following wine analytical methodologies.

**Table 1:** **Fining agents and respective dosages tested**.

**Fining products**	**Minimum dosage (g/hL)**	**Maximum dosage (g/hL)**
Bentonite	10	60
PVPP	10	80
Casein	20	100
Vegetable protein	20	60
BCV1	10	20
BCV5	10	20

#### After oxidation during 5 months

A volume of 5 L from each untreated wine barrel was purposely reserved in the large wine vessels (20 L) and stored at room temperature during 5 months. During this period, wines had natural contact with oxygen and no additional rectification with sulfur dioxide was performed. After 5 months, a second experiment of fining trials was performed, on the resultant highly oxidized and turbid wines. Again, the fining trials were conducted during 48 h, but this time in glass bottles of 550 ml of total volume. Each glass bottle was filled in with 500 ml of wine and the assays performed in duplicates, in this case, only testing the effect of YPE (BCV1 and BCV5) and casein. After 48 h fining, wine samples were processed according to the methodology previously described (Section After Alcoholic Fermentation)

#### Protein fining agents characterization

As described by Vincenzi et al. (2005), a 10% stock solution of sodium-dodecyl sulfate (SDS) was prepared and then added to wine to achieve final concentrations of 0.1%. Samples were gently mixed during 2 min and then heated in a boiling water bath for 5 min. Potassium chloride (KCl) (2 M) was added to each sample to attain a final concentration of 200 mM. Samples were set to incubate for 1 h and KDS-protein pellets were recovered by centrifugation at 14,000 g for 15 min at 4°C (Vincenzi et al., 2005). Pellets were further dissolved in 1× phosphate buffered saline, pH 7.4 (PBS buffer). Protein content (%w/w) was further accessed using BCA Assay kit from Pierce. Further, the protein molecular weight profile of each protein fining agent was acquired by SDS-PAGE electrophoresis in concordance with the OIV resolution [Resolution OIV-OENO 452-2012]. Gel electrophoresis was performed on a Bio-Rad Protean II apparatus with power supply set at 100V/gel for the stacking gel and 150V/gel for the resolving gel. Protein samples were equally prepared in Laemmli Sample Buffer (5X) and boiled at 95°C during 5 min. 12.5% polyacrylamide resolving gels were used to process the samples and the gel was afterward stained using Coomassie Blue R250 reagent. The isoelectric point of the protein fining agents and YPE was measured using the Stabino Charge Titration System, measured in triplicates.

### Wine analysis

#### Conventional oenological parameters

Free and total SO_2_, density, alcohol content, titratable acidity (TA), volatile acidity (VA), malic acid, tartaric acid, pH, and glycerol content were controlled using a FOSS Wine-Scan (FT-120) infrared Fourier-transform spectrometer and a WineScan software Version 2.2.1 (FOSS, Hillerod, Denmark). The phenolic content was expressed as Total Phenolic Index at 280 nm (TPI) according to the O.I.V. method n. ENO/SCMAV/04/298 (2006). According to Batista et al. (2009), final turbidity was measured at 540 nm in 1-cm path length quartz cells (Batista et al., 2009).

#### Lees volume

Lee volume was acquired by directly measuring the thickness of the sediment in the glass bottles after the fining trials were completed. Results were expressed as percentage of the initial volume of wine (% v/v).

#### Chromatic characteristics

The absorption spectrum of each wine was recorded through a spectrophotometry methodology in a Cary 100 BIO UV-Visible spectrophotometer, using the Cary WinUV Scan software. Absorbance was scanned for each sample over the range of 280–750 nm, using quartz cells of 1-cm path length. The absorbance values were recorded at 10 nm intervals. Following, the chromatic coordinates of C^*^ (chroma or “saturation”), L^*^ (lightness or “brilliance”), a^*^ (redness), and b^*^ (yellowness) were calculated using the CIELab system in the MSCV^®^ software, from Grupo de Color de La Rioja, Logroño, Spain) (Perez-Magarino and Jose, 2002).

#### Wine unstable proteins

Protein content present in wine samples was measured by BCA kit methodology (Bio-Rad) using bovine serum albumin as the standard. The protein molecular weight pattern of each wine sample was assessed by SDS-PAGE electrophoresis, performed after protein precipitation with the KDS-method, according to Vincenzi et al. (2005).

#### Heat instability

Heat instability was accessed by the heat test methodology described in other studies (Fusi et al., 2010; Benucci et al., 2014) and also recommended by OIV (OIV-Oeno 494-2012) with small modifications. In brief, wine samples of 10 ml volume were set in test tubes and closed using screw caps. The tubes were heated at 80°C in a water bath for 30 min and then allowed to cold to room temperature during 2 h. The increase in turbidity was detected by spectrophotometry (Cary 100 BIO UV-Visible spectrophotometer and Cary WinUV software) at 540 nm in 1 ml quartz cuvettes and expressed as Δ*A*_540_nm. All measurements were performed in triplicate.

#### Browning prevention effect: forced and natural oxidation

Browning prevention was tested in two separated assays. In the first test, browning potential was measured by submitting the wine samples to oxidize during 3 days. Two different tubes were prepared for each wine sample: In tube A (control), 15 ml of each wine sample was introduced into a 15 ml falcon tubes and carefully nitrogen-sparged during 2 min. In tube B, 10 ml of each wine sample was introduced into a 15 ml falcon tubes and 3% (w/v) hydrogen peroxide (Merck, Millipore) was added, without nitrogen addition (Cosme et al., 2008). All the tubes were left to incubate at room temperature during 3 days. In a separated experiment, the natural oxidation of the wines was also tested. This test was performed by simply leaving the wines treated with YPE and casein in totally filled glass bottles of 200 ml volume during 5 and 9 months. For both tests, the browning variation was accessed by measuring the optical density variation at 420 nm (Δ*A*_420_nm).

#### Curative test

For this experiment, the three base wines were stored to naturally oxidize during 5 months. After that period, the oxidized wines were treated with YPE and casein in 500 ml fining assays (as referred in topic After Oxidation during 5 Months). After fining, wine samples were analyzed by CIELab methodology (as presented in Chromatic Characteristics) and the achievements compared with the first fining trials, performed after alcoholic fermentation.

### Statistical analysis

Statistical analysis was performed in Primer software, version 6.1.16 (Clarke and Gorley, 2006). Standard deviation values calculations and One-Way ANOVA tests were performed. Differences of *p* < 0.05 were considered significant.

## Results and discussion

### Protein fining agents

**Figure 1:**
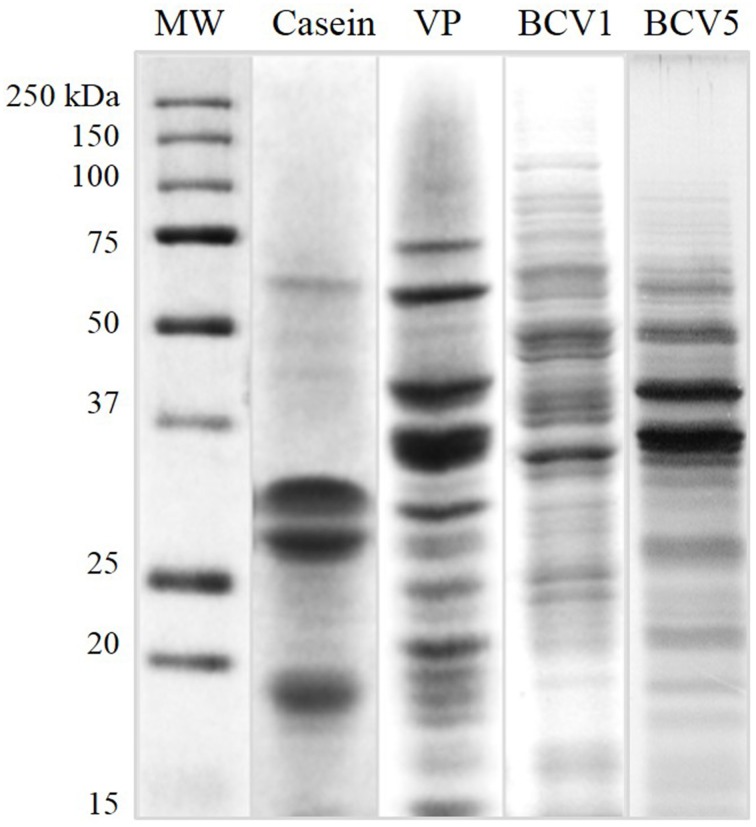
**Protein molecular weight profile**. Protein samples of the fining agents used in this study by Comassie-stained SDS-PAGE (VP, Vegetable protein).

The physicochemical characteristics of the protein fining agents used in this study are presented in Table 2. Both YPE (BCV1 and BCV5) presented isoelectric point values (*pI* = 4.4 and 4.5) close to casein (*pI* = 4.6). In terms of protein composition, YPE presented protein contents (%w/w) above 50% as required in the OIV regulation regarding YPE fining (OIV-Oeno 494-2012). The low protein content presented by the vegetable protein fining agent, might indicate that this product probably contains other fining compounds on its formulation. In addition, SDS-PAGE analysis (Figure 1) shows that 50% of the total yeast protein is located above 15 kDa of molecular weight, which is also in accordance with the OIV demand. The distribution of the protein bands was observed between the molecular weight range of 10–150 kDa and the electrophoretic profile revealed low signs of protein degradation. Proteins detected at 48 kDa might correspond enolases (ENO1 and ENO2).

**Table 2:** **Fining agents used with the respective physicochemical characteristics**.

**Fining products**	**Isoelectric point (mean ± SD)**	**Protein content (% w/w)**
Bentonite	−	−
PVPP	−	−
Casein	4.6 ± 0.1	65.3
Vegetable protein	5.2 ± 0.0	19.5
BCV1	4.5 ± 0.2	62.4
BCV5	4.4 ± 0.1	60.1

Casein presented intense individual bands located at 18, 30, and 34 kDa, probably respectively corresponding to the whey protein β-lactalbumin, α_*s*_-Casein subunit, and β-Casein subunit (Gambuti et al., 2012; Fleminger et al., 2013). Regarding the vegetable protein (VP) from gluten origin, the protein bands varied from 10 to 70 kDa of molecular weight. This protein profile corresponds, as expected, to hydrolyzed wheat proteins and the different bands correspond to distinct glutenin subunits (Chinuki et al., 2013).

### Clarification potential

#### Final turbidity

**Figure 2:**
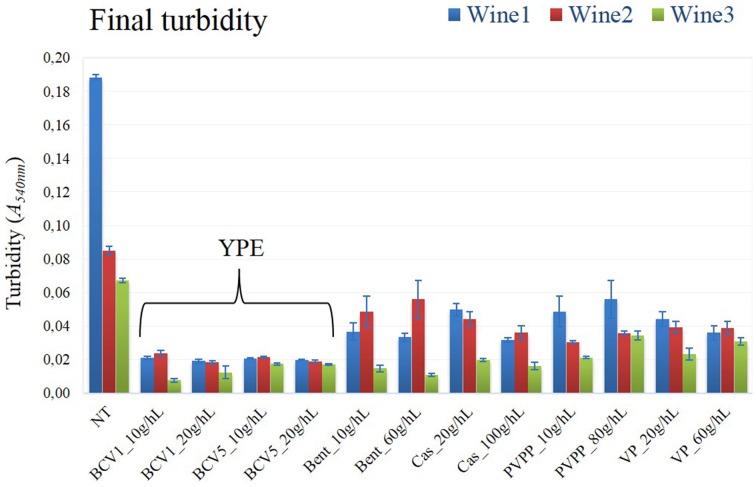
**Final turbidity**. Not treated wine, NT; Treated wine samples, Yeast protein extracts (BCV1 and BCV5); Bent, Bentonite; Cas, Casein; PVPP, Polyvinylpolypyrrolidon; VP, Vegetable protein. Bars indicate mean ± SD (*n* = 3).

After the fining trial performed in 250 ml glass bottles, the impact of the YPE and reference fining agents was first compared. No significant differences were detected regarding the conventional oenological parameters accessed by infrared Fourier-transform spectrometer (Supplementary material). Contrarily, YPE revealed a superior capacity to reduce turbidity, comparing with the efficiency of all the reference fining agents tested in this study (Figure 2). Indeed, both extracts shown ability to clarify the most turbid wines as Wine 1 (reduction of 89%) and Wine 2 (76% reduction), but also to reduce the turbidity of the least turbid wine (79% reduction). In agreement to this, previous studies concerning the application of reference fining agents from animal, mineral, and vegetable origins show comparable or inferior percentages of turbidity reduction when compared with the effect of our YPE (Bonilla et al., 2001; Marchal et al., 2002; Braga et al., 2007; Sauvage et al., 2010; Lucchetta et al., 2013). Other study tested the effect of YPE in red wines and also proved their ability to reduce wine turbidy to values that were comparable to gelatins (Iturmendi et al., 2010). Regarding the three different wines tested in this study, the final turbidity values obtained by YPE fining were located in a range of Abs*A*_540_nm between 0.007 and 0.023 a.u. (absorbance units), a result that could not be obtained by the application of any reference fining agent herein tested. It is important to remark that the maximum dosage used for YPE (20 g/hL) was 3–5 times smaller than the reference products (60–100 g/hL).

#### Color characterization

**Figure 3:**
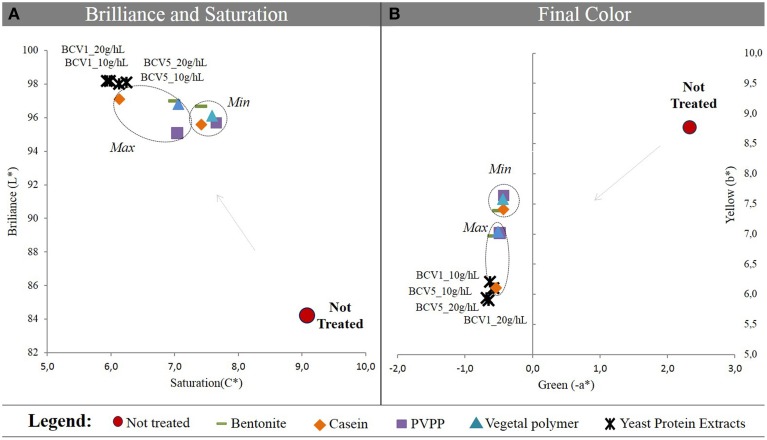
**Chromatic characterization using CIELab system**. **(A)** Saturation (C^*^), Brilliance (L^*^); **(B)** Green (-a^*^), and Yellow (b^*^) values. Results were obtained before and after treatment of wine 1 with YPE and different fining agents. Two dosages were tested by treatment (Min, minimum; Max, maximum).

As shown in Figure 3A the CIELab methodology used in this study revealed that when Wine 1 was treated with YPE presented a superior increase of brilliance (L^*^), along with a superior reduction of saturation (C^*^). Color improvement was also verified for the wine treated with both yeast extracts, when compared to the reference fining agents. The final samples were found to be more greenish (−a^*^) and simultaneously less yellowish (b^*^), when compared with the other wine samples Figure 3B. This also demonstrated that YPE have the ability to remove undesired yellow pigments contained in the wines matter and that could cause oxidative problems after bottling. Only by applying the maximum dosage of casein comparable results were achieved and this could indicate that YPE might represent a good alternative to this animal origin product. CIELab data is in agreement with the results obtained in terms of turbidity reduction and were also confirmed in the three different wine patterns tested in this study. See supplementary material to consider the results obtained in wine 2 and 3.

#### Lees volume

**Figure 4:**
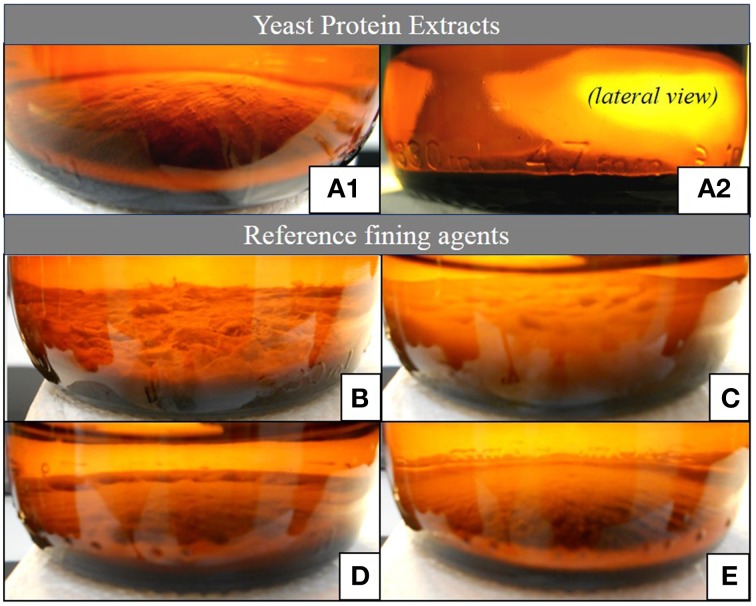
**Aspect of the lees produced by the application YPE or fining agents**. (**A.1,A.2**) Yeast protein extract BCV1, similar result was obtained with BCV5; **(B)** Casein, 100 g/hL; **(C)** Bentonite, 60 g/hL; **(D)** PVPP, 80 g/hL; **(E)** Vegetable polymer, 60 g/hL.

As presented in Figure 4, the lees formed after the YPE application were visually more compact and homogeneous than the lees obtained by using of the reference fining agents. In terms of final volume and homogeneity, the YPE lees were found to be similar with those obtained with vegetable protein application. Accordingly, the volume percentage measured in relation to the wine initial volume was approximately 22, 31, and 44% smaller than the lees respectively obtained with casein, PVPP, and bentonite application (Table 3). This evidence is in agreement with the results reported by Iturmendi et al. (2010) in red wines fining, showing a correlation between lower lees volume and the proximity of protein Ip to a value of 4.43 (isoelectric point of: BCV1 = 4.5 ± 0.2; BCV5 = 4.4 ± 0.2). This study also refers that YPE produced smaller lees in red wines fining when compared with the impact of gelatin (Iturmendi et al., 2010). The final aspect and thickness of the lees produced by YPE increase their potential use as alternative clarification agents, since are appreciated characteristics for the industrial clarification process implementation. Overall, YPE seem to be promising technological tools that contribute to prepare wine for filtration and also reduce the wine loss on the vats bottom. In addition, since they are harmless and biodegradable, have no constrains in terms of process handling or dregs disposal, contrary to bentonite, or PVPP.

**Table 3:** **Lee volume percentage values obtained after fining trials with the different fining agents and YPE**.

**Lees volume (% v/v) Mean ± SD**		
**Fining products**	**Min. dosage**	**Max. dosage**
Bentonite	5.91% ± 0.12	6.87% ± 0.00
PVPP	5.00% ± 0.10	5.71% ± 0.01
Casein	4.29% ± 0.05	5.00% ± 0.05
Vegetable protein	2.86% ± 0.00	3.75% ± 0.02
BCV1	3.37% ± 0.02	3.53% ± 0.02
BCV5	3.42% ± 0.02	4.09% ± 0.05

*Mean and standard deviation values were calculated concerning the three wines*.

### Stabilization potential

#### Protein haze protection

**Figure 5:**
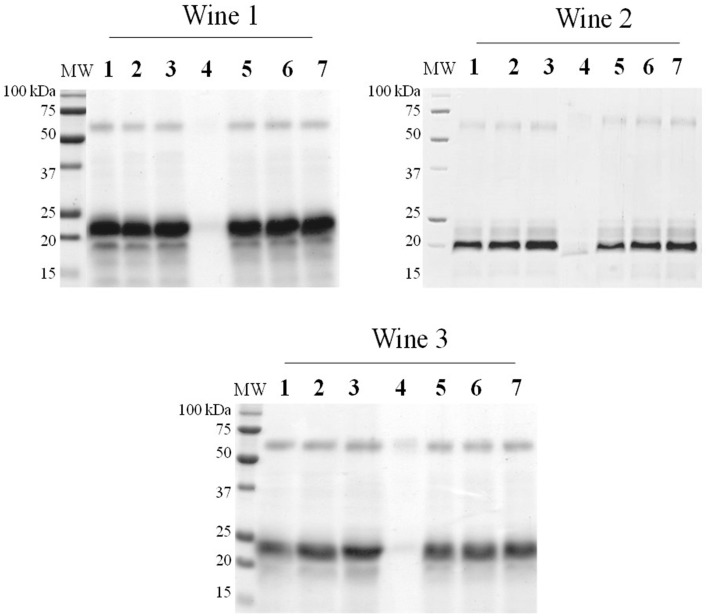
**SDS-PAGE 12,5%, Comassie staining**. Total protein was precipitated from 1 ml of each wine sample. 1, Not treated wine. Wines treated with fining agents (max. dosage); 2, BCV1; 3, BCV5; 4, Bentonite; 5, Casein; 6, PVPP; 7, Vegetable protein.

**Figure 6:**
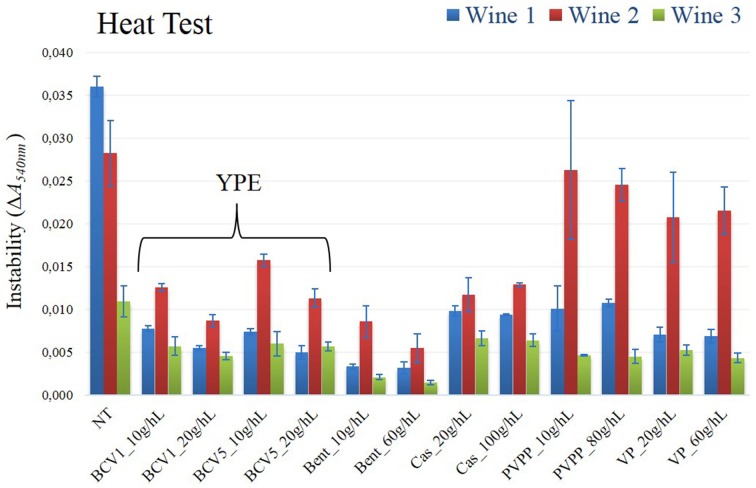
**Heat test**. Instability was measured according to the variation of absorbance at 540 nm, by spectrometry methodology (Δ*A*_540_nm). Not treated wine, NT; Treated wine samples, Yeast protein extracts (BCV1 and BCV5); Bent, Bentonite; Cas, Casein; PVPP, Polyvinylpolypyrrolidon; VP, Vegetable protein. Bars indicate mean ± SD (*n* = 3).

The results of the SDS-PAGE analysis performed in the wine samples show, as expected, that unstable proteins, specifically, *thaumatin-like proteins* (MW ~20–25 kDa) and*chitinases* (MW ~32 kDa), have only been removed from the wines treated with bentonite (Figure 5). By having an isoelectric point superior to wine pH, the yeast extracts became positively charge when added to wine and therefore, they were not able to interact with the positively charged unstable proteins, contrarily to the negatively charged bentonite clay. (Pocock et al., 2000; Marangon et al., 2011). Moreover, no bands of residual fining yeast proteins were detected in SDS-Page gels, which might indicate that the majority of the yeast proteins have been precipitated. This evidence correlates with the clarification potential results previously presented in this study, which indicate that flocculation seems to have occurred very efficiently. Nevertheless, a more sensitive method as ELISA or mass spectrometry methodologies should be further used to analyze the probable presence of some residual yeast proteins on the final wines. Although the wine unstable proteins have not been removed by the YPE fining, the results of the heat test indicate that heat instability was significantly reduce (*p* < 0.05) after the fining treatment in comparison to the untreated wines (Figure 6). Data obtained in wines treated with YPE and casein were not significantly different (*p* < 0.05) and as expected, bentonite was the fining agent that promoted the higher instability reduction due to its capacity to flocculate the problematic wine unstable proteins. A recent study also tested the effect of reference fining agents on white wines protein haze and demonstrate that casein, egg albumin, isinglass, chitosan, chitin, and PVPP did not significantly affect the tendency of the wine to form protein haze (Chagas et al., 2012). Although the haze protection mechanism is still very unclear in the literature (Moine-Ledoux and Dubourdieu, 1999), the stabilization effect promoted by YPE application is possibly explained by the presence of some residual haze-protective material on our extracts. In particular it is probable that the YPE still contain mannoproteins (glycoproteins containing 15–90% mannose by their weight) derived from the yeast cell walls which are already well described to prevent the haze phenomenon in white wines (Dupin et al., 2000a,b; Caridi, 2006). Indeed, some studies previously showed that wine aged on yeast lees presented reduced haze potential and lower bentonite requirements for protein stability than wine aged without lees but containing the same level of unstable protein (Moine-Ledoux and Dubourdieu, 1999). In addition, mannoproteins might also promote a positive impact in terms of wine body and volume enhancement (Caridi, 2006).

#### Browning prevention

**Figure 7:**
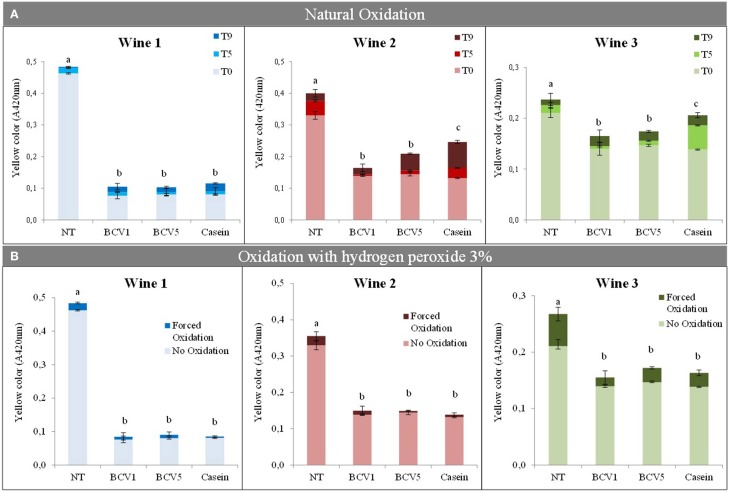
**Browning evaluation after natural and forced wine oxidations. (A)** Yellow color (expressed as *A*_420_nm) was measured after 0, 5, 9 months of oxidation, respectively: T0, T5, T9. **(B)** Oxidation was evaluated before and after oxidation using a solution of 3% (w/v) hydrogen peroxide. Different letters represent significant differences (*p* < 0.05). Bars indicate mean ± SD (*n* = 3).

Tests were performed to understand the tendency of the untreated and treated wines to oxidize. Casein was used as control to these tests, since it is a phenolic reactive agent often used to reduce oxidizable flavonoid and non-flavanoid fractions, and therefore to reduce the probability of the wine to oxidize (Schneider, 1995; Cosme et al., 2012). In the first test, wines were stored under natural oxidative conditions, without sulfur dioxide supplementation, during 5 and 9 months. Results show that YPE were able to prevent the three wines to naturally oxidize (Figure 7). Their efficiency was significantly (*p* < 0.05) similar or in some cases superior when compared with the results obtained by using casein. In particular, wines 2 and 3 became significantly less oxidized with YPE treatment than the ones treated with casein after the 9 months. In order to compare two distinct procedures, the untreated and treated wines were also submitted to the presence of a strong oxidizer, 3% (w/v) hydrogen peroxide during 3 days. In this case, results revealed no significant differences (*p* < 0.05) between the impact obtained with YPE or casein. Both treatments herein presented seem to be efficient in preventing the browning phenomenon even after contact with extreme oxidation conditions. Wines 2 and 3 showed to be the more susceptive to the browning phenomenon.

#### Curative capacity

**Figure 8:**
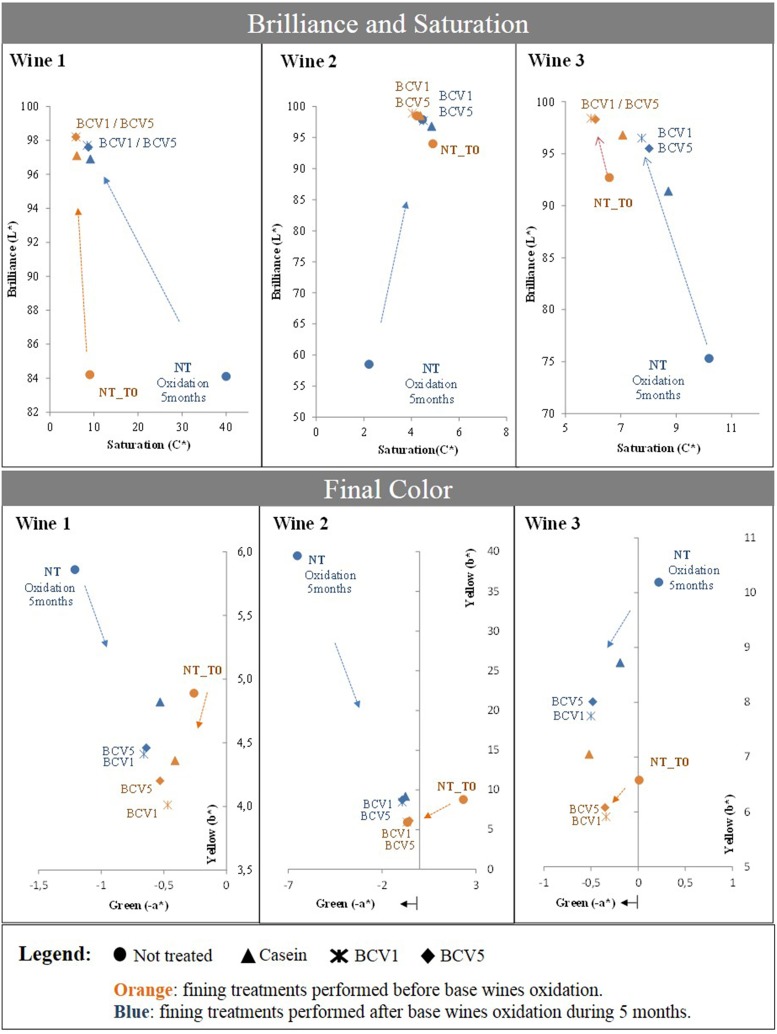
**Test of curative potential using chromatic characterization**. Not treated samples were fined after 5 months oxidation and effects were compared with the previously obtained results (T0). Arrows indicate the impact obtained after the respective treatments.

In Figure 8 a comparison is presented between the effects of the fining treatments performed in the wines received after alcoholic fermentation (T0) and fining treatments regarding the intensively oxidized wines (after 5 months - browning). In both fining trials, YPE promoted similar tendencies and achievements toward brilliance and color improvement, proving that they efficiently correct wine browning, independently from the oxidation starting point or pattern of each wine. For both experiments, CIELab indexes show increase of Brilliance (L^*^) to values above 95 units along with saturation reduction to values below 10 units. In parallel, YPE treatment promoted a clear yellow color reduction (b^*^) especially obvious in the extremely oxidized wines, 1 and 2. Moreover, a parallel increase in terms of green (-a^*^) was verified in the case of the oxidized wine 3. Although browning is normally difficult to correct in excessively oxidized wines, casein is recognized by its curative capacity to decolor the oxidized wines, contributing to color refreshment while also refining gustatory characteristics (Braga et al., 2007). In comparison to casein, YPE revealed superior capacity to correct the browning regarding the three wines and with both dosages tested in our study (10 and 20 g/hL). Bonilla et al. (2001) previously tested a correction treatment using baker's yeasts in white wines and reported that the use of yeast at 1 g/L as a fining treatment was able to correct browning with similar results in terms of *A*_420_nm decrease in comparison with a traditional treatment based on activated charcoal application (Bonilla et al., 2001). In agreement with this fact, other study revealed that whole yeasts or yeast cell walls present the capacity to efficiently adsorb phenolic compounds and browning products and thus decrease the yellow–brown color levels after white wines fining (Razmkhab et al., 2002). Overall, our data suggests that YPE also have a preventive and curative capacity to correct white wines. Similarly to casein, yeast extracts might probably have the capacity to remove some oxidizable and oxidized species as phenolic compounds, in particular flavan-3-ol derivates, considered primarily responsible for oxidative aging of white wines, since small amounts of these molecules might generate browning (Schneider, 1995). The presence of other compounds as iron, copper, acetaldehyde, or tartaric acid might also contribute to accelerate different types of condensation and oxidative reactions, contributing to the production brown compounds (Razmkhab et al., 2002). Based on this, further research is necessary to better understand the protective and corrective mechanism behind the YPE fining potential.

## Conclusions

In this work we revealed the potential of novel YPE toward white wine clarification, stabilization, and curative processes. Particularly, two YPE previously selected and optimized for this study, were able to promote a significant brilliance increase, turbidity reduction and final color improvement when compared with the reference fining agents available on the market.

The lees produced after the YPE treatment were indeed found to be more compact and thick than the obtained with reference formulations, conferring to this yeast by-products an extra technological advantage in terms of the industrial fining validation. A protective effect to protein haze was also verified in samples fined with YPE, although they were not able to interact and remove unstable proteins as bentonite did. YPE treatment also revealed a superior effect on browning prevention and curative ability when compared to casein, an animal origin product, already well-recognized in the literature to efficiency reduce oxidation and browning in white wines (Cosme et al., 2008).

Overall, in this study we present an innovative fining alternative that can efficiently contribute to improve white wines final quality. Indeed, YPEs represent a novel tool for the wine sector, since they are obtained from a completely native source. This treatment represents a more sustainable alternative when compared to the exogenous substances from mineral, animal proteins or wheat origins. In future work, is our aim to further investigate the impact of these YPE in terms of sensorial analysis and to test their potential implementation at industrial scale.

### Conflict of interest statement

The authors declare that the research was conducted in the absence of any commercial or financial relationships that could be construed as a potential conflict of interest.
