# Corrigendum: Unveiling the potential of novel yeast protein extracts in white wines clarification and stabilization

**DOI:** 10.3389/fchem.2015.00031

**Published:** 2015-05-08

**Authors:** Joana P. Fernandes, Rodrigo Neto, Filipe Centeno, Pedro Castanheira, Maria De Fátima Teixeira, Ana C. Gomes

**Affiliations:** ^1^Genomics Unit, BiocantCantanhede, Portugal; ^2^PROENOL-Indústria Biotecnológica, Lda.Canelas, Portugal; ^3^Molecular Biotechnology Unit, BiocantCantanhede, Portugal

**Keywords:** corrigendum, authorship, authors, alteration, contribution

The list of authors of this Article should be corrected in order to include Dr. Pedro Castanheira. Therefore, the authorship order should be considered as follows:

Joana P. Fernandes^1^, Rodrigo Neto^1^, Filipe Centeno^2^, Pedro Castanheira^3^, Maria De Fátima Teixeira^2^ and Ana Catarina Gomes^1*^.

1. ^1^ Genomics Unit, Biocant, Núcleo 4, Lote 8, 3060-197 Cantanhede, Portugal; e-mails: joana.fernandes@biocant.pt; rodrigo.neto@biocant.pt; acgomes@biocant.pt
2. ^2^ PROENOL—Indústria Biotecnológica, Lda, Travessa das Lages n° 267, Apto 547, Canelas, VNG 13 4405-194 Portugal; e-mails: filipe.centeno@proenol.pt; maria.fatima@proenol.pt
3. ^3^ Molecular Biotechnology Unit, Biocant, Núcleo 4, Lote 8, 3060-197 Cantanhede, Portugal; e-mails: pedro.castanheira@biocant.pt

Pedro Castanheira has actively participated on the experimental design and optimization of the protein extraction procedures, contributing to the development of the referred yeast protein extracts.

The authors regret the error.

## Conflict of interest statement

The authors declare that the research was conducted in the absence of any commercial or financial relationships that could be construed as a potential conflict of interest.

