# Supplementary material for: Unveiling the potential of novel yeast protein extracts in white wines clarification and stabilization
Source: Front Chem. 2015 Mar 18;3:20. doi: 10.3389/fchem.2015.00020 (PMC4364169; doi:10.3389/fchem.2015.00020)
Supplement: Supplementary file 1 [file Presentation1.PPTX]

## Slide 1
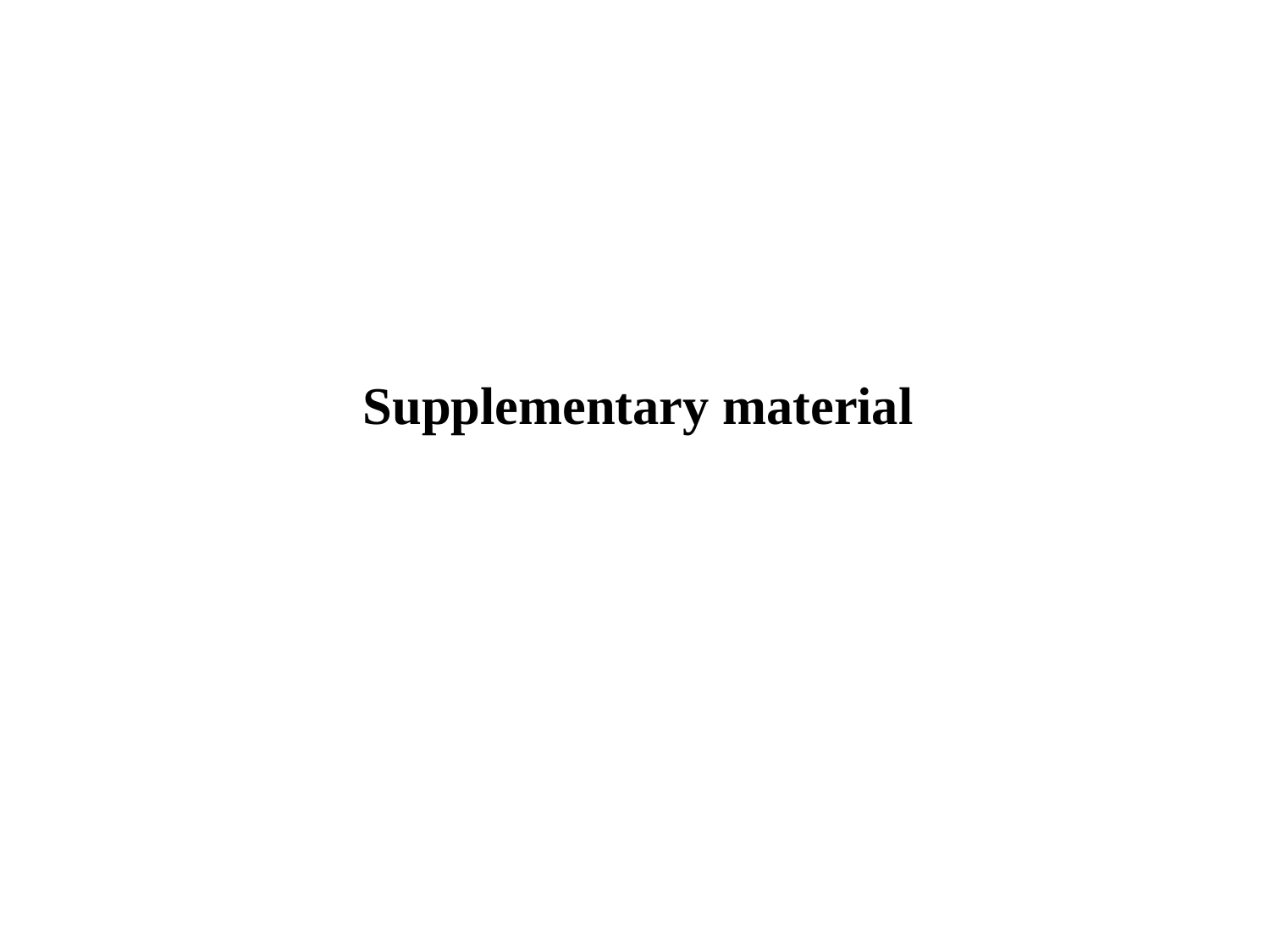

Supplementary material

## Slide 2
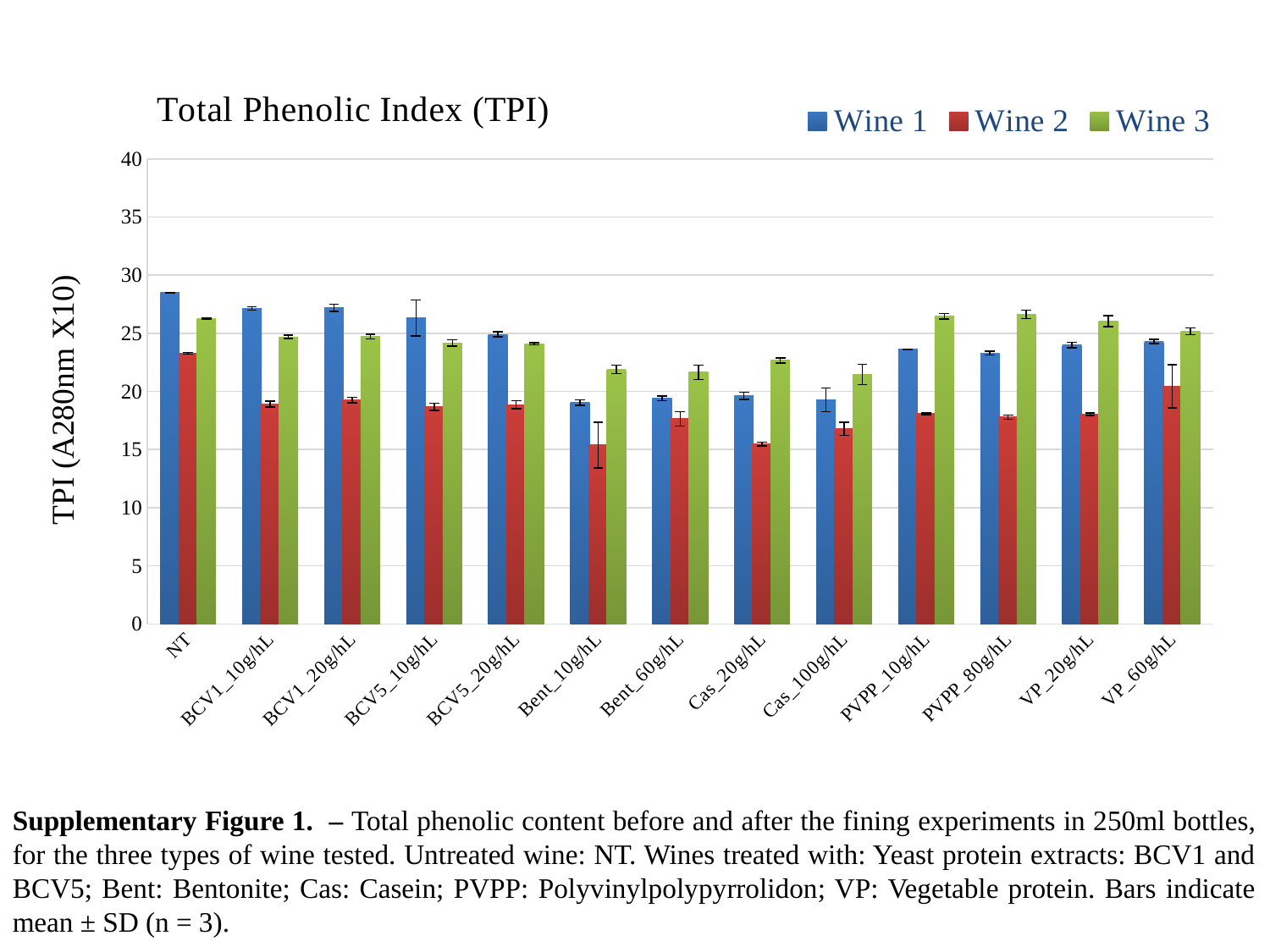

### Chart: Total Phenolic Index (TPI)
| Category | | | |
|---|---|---|---|
| NT | 28.5 | 23.27 | 26.26 |
| BCV1_10g/hL | 27.136666666666667 | 18.91333333333333 | 24.69333333333333 |
| BCV1_20g/hL | 27.189999999999998 | 19.26 | 24.709999999999997 |
| BCV5_10g/hL | 26.313333333333333 | 18.683333333333334 | 24.166666666666668 |
| BCV5_20g/hL | 24.916666666666668 | 18.866666666666667 | 24.110000000000003 |
| Bent_10g/hL | 19.036666666666665 | 15.386666666666665 | 21.893333333333334 |
| Bent_60g/hL | 19.41 | 17.636666666666667 | 21.653333333333336 |
| Cas_20g/hL | 19.633333333333333 | 15.469999999999999 | 22.663333333333338 |
| Cas_100g/hL | 19.276666666666667 | 16.786666666666665 | 21.459999999999997 |
| PVPP_10g/hL | 23.64 | 18.083333333333332 | 26.47333333333333 |
| PVPP_80g/hL | 23.293333333333333 | 17.786666666666665 | 26.636666666666667 |
| VP_20g/hL | 23.98 | 18.05 | 26.05 |
| VP_60g/hL | 24.290000000000003 | 20.426666666666666 | 25.163333333333338 |Supplementary Figure 1. – Total phenolic content before and after the fining experiments in 250ml bottles, for the three types of wine tested. Untreated wine: NT. Wines treated with: Yeast protein extracts: BCV1 and BCV5; Bent: Bentonite; Cas: Casein; PVPP: Polyvinylpolypyrrolidon; VP: Vegetable protein. Bars indicate mean ± SD (n = 3).

## Slide 3
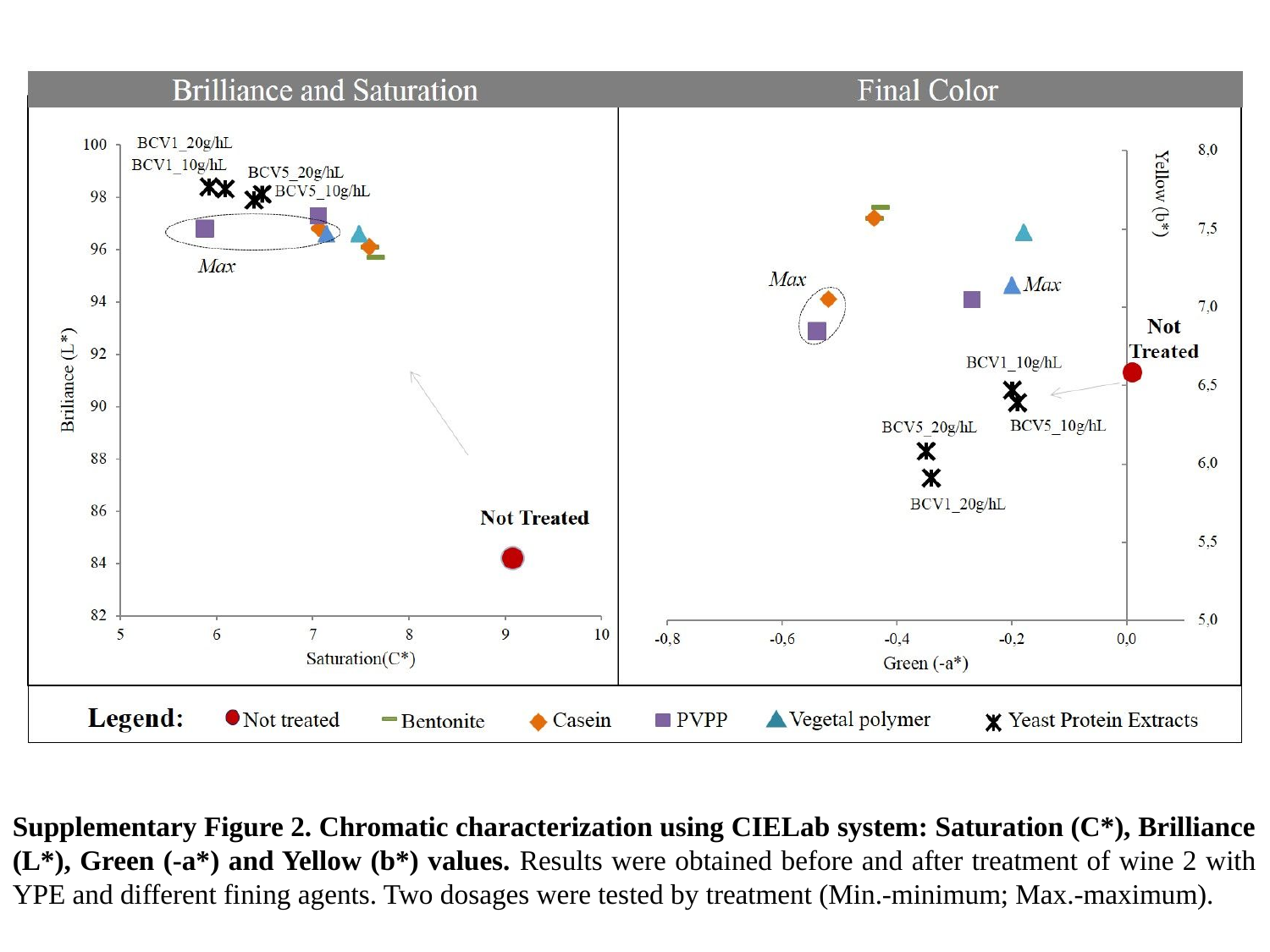

Supplementary Figure 2. Chromatic characterization using CIELab system: Saturation (C*), Brilliance (L*), Green (-a*) and Yellow (b*) values. Results were obtained before and after treatment of wine 2 with YPE and different fining agents. Two dosages were tested by treatment (Min.-minimum; Max.-maximum).

## Slide 4
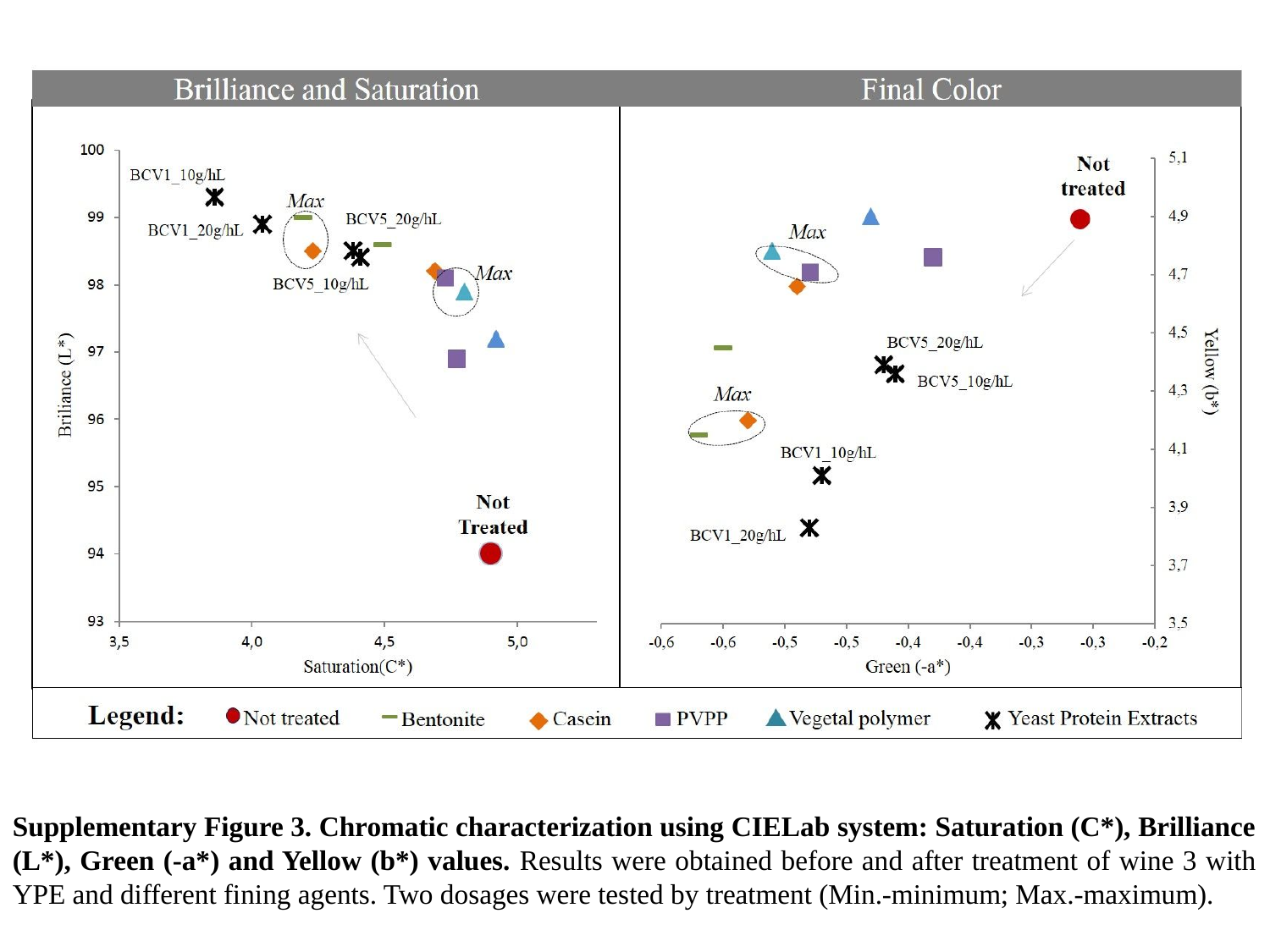

Supplementary Figure 3. Chromatic characterization using CIELab system: Saturation (C*), Brilliance (L*), Green (-a*) and Yellow (b*) values. Results were obtained before and after treatment of wine 3 with YPE and different fining agents. Two dosages were tested by treatment (Min.-minimum; Max.-maximum).

## Slide 5
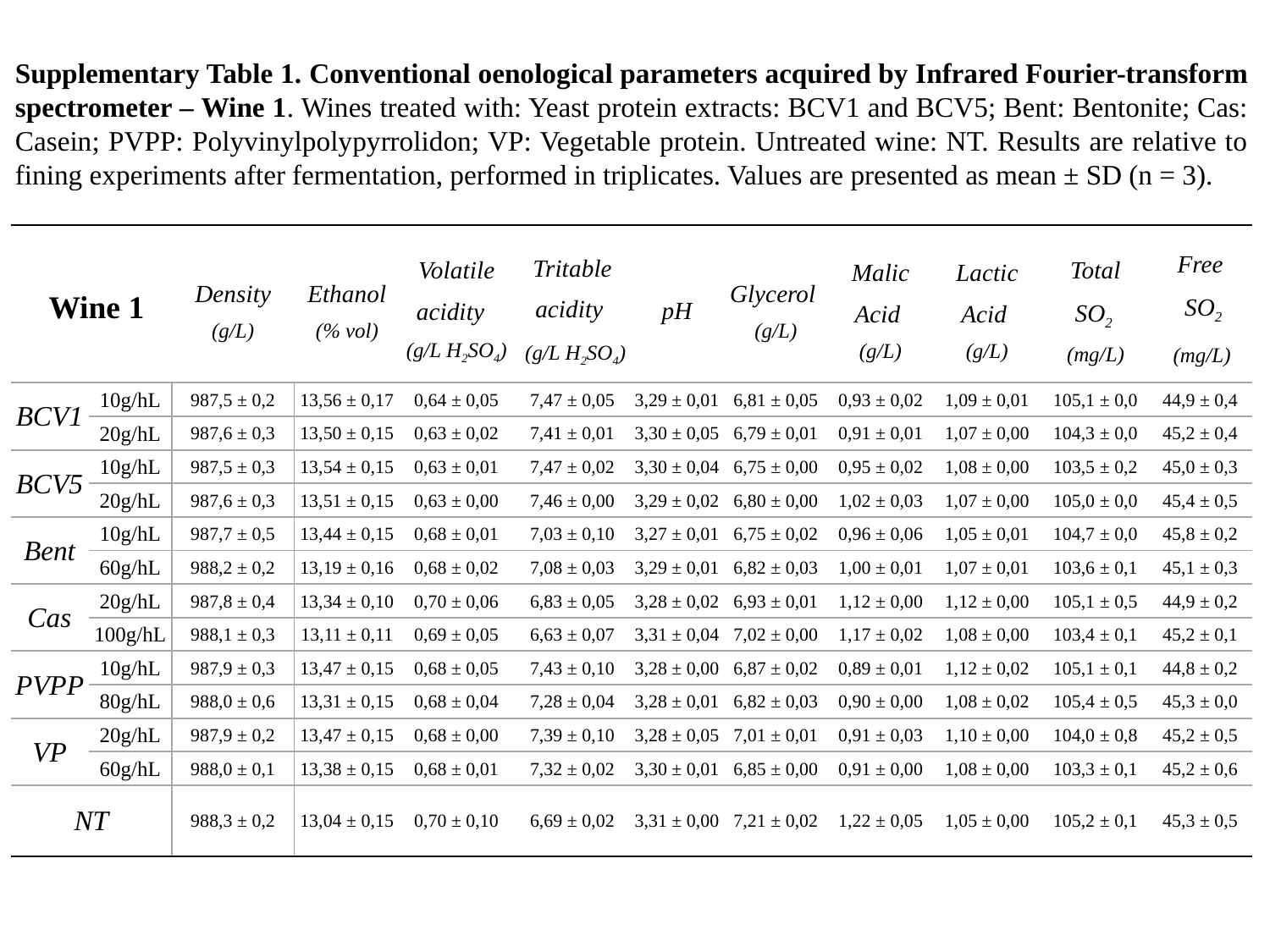

Supplementary Table 1. Conventional oenological parameters acquired by Infrared Fourier-transform spectrometer – Wine 1. Wines treated with: Yeast protein extracts: BCV1 and BCV5; Bent: Bentonite; Cas: Casein; PVPP: Polyvinylpolypyrrolidon; VP: Vegetable protein. Untreated wine: NT. Results are relative to fining experiments after fermentation, performed in triplicates. Values are presented as mean ± SD (n = 3).
| Wine 1 | | Density (g/L) | Ethanol (% vol) | Volatile acidity (g/L H2SO4) | Tritable acidity (g/L H2SO4) | pH | Glycerol (g/L) | Malic Acid (g/L) | Lactic Acid (g/L) | Total SO2 (mg/L) | Free SO2 (mg/L) |
| --- | --- | --- | --- | --- | --- | --- | --- | --- | --- | --- | --- |
| BCV1 | 10g/hL | 987,5 ± 0,2 | 13,56 ± 0,17 | 0,64 ± 0,05 | 7,47 ± 0,05 | 3,29 ± 0,01 | 6,81 ± 0,05 | 0,93 ± 0,02 | 1,09 ± 0,01 | 105,1 ± 0,0 | 44,9 ± 0,4 |
| | 20g/hL | 987,6 ± 0,3 | 13,50 ± 0,15 | 0,63 ± 0,02 | 7,41 ± 0,01 | 3,30 ± 0,05 | 6,79 ± 0,01 | 0,91 ± 0,01 | 1,07 ± 0,00 | 104,3 ± 0,0 | 45,2 ± 0,4 |
| BCV5 | 10g/hL | 987,5 ± 0,3 | 13,54 ± 0,15 | 0,63 ± 0,01 | 7,47 ± 0,02 | 3,30 ± 0,04 | 6,75 ± 0,00 | 0,95 ± 0,02 | 1,08 ± 0,00 | 103,5 ± 0,2 | 45,0 ± 0,3 |
| | 20g/hL | 987,6 ± 0,3 | 13,51 ± 0,15 | 0,63 ± 0,00 | 7,46 ± 0,00 | 3,29 ± 0,02 | 6,80 ± 0,00 | 1,02 ± 0,03 | 1,07 ± 0,00 | 105,0 ± 0,0 | 45,4 ± 0,5 |
| Bent | 10g/hL | 987,7 ± 0,5 | 13,44 ± 0,15 | 0,68 ± 0,01 | 7,03 ± 0,10 | 3,27 ± 0,01 | 6,75 ± 0,02 | 0,96 ± 0,06 | 1,05 ± 0,01 | 104,7 ± 0,0 | 45,8 ± 0,2 |
| | 60g/hL | 988,2 ± 0,2 | 13,19 ± 0,16 | 0,68 ± 0,02 | 7,08 ± 0,03 | 3,29 ± 0,01 | 6,82 ± 0,03 | 1,00 ± 0,01 | 1,07 ± 0,01 | 103,6 ± 0,1 | 45,1 ± 0,3 |
| Cas | 20g/hL | 987,8 ± 0,4 | 13,34 ± 0,10 | 0,70 ± 0,06 | 6,83 ± 0,05 | 3,28 ± 0,02 | 6,93 ± 0,01 | 1,12 ± 0,00 | 1,12 ± 0,00 | 105,1 ± 0,5 | 44,9 ± 0,2 |
| | 100g/hL | 988,1 ± 0,3 | 13,11 ± 0,11 | 0,69 ± 0,05 | 6,63 ± 0,07 | 3,31 ± 0,04 | 7,02 ± 0,00 | 1,17 ± 0,02 | 1,08 ± 0,00 | 103,4 ± 0,1 | 45,2 ± 0,1 |
| PVPP | 10g/hL | 987,9 ± 0,3 | 13,47 ± 0,15 | 0,68 ± 0,05 | 7,43 ± 0,10 | 3,28 ± 0,00 | 6,87 ± 0,02 | 0,89 ± 0,01 | 1,12 ± 0,02 | 105,1 ± 0,1 | 44,8 ± 0,2 |
| | 80g/hL | 988,0 ± 0,6 | 13,31 ± 0,15 | 0,68 ± 0,04 | 7,28 ± 0,04 | 3,28 ± 0,01 | 6,82 ± 0,03 | 0,90 ± 0,00 | 1,08 ± 0,02 | 105,4 ± 0,5 | 45,3 ± 0,0 |
| VP | 20g/hL | 987,9 ± 0,2 | 13,47 ± 0,15 | 0,68 ± 0,00 | 7,39 ± 0,10 | 3,28 ± 0,05 | 7,01 ± 0,01 | 0,91 ± 0,03 | 1,10 ± 0,00 | 104,0 ± 0,8 | 45,2 ± 0,5 |
| | 60g/hL | 988,0 ± 0,1 | 13,38 ± 0,15 | 0,68 ± 0,01 | 7,32 ± 0,02 | 3,30 ± 0,01 | 6,85 ± 0,00 | 0,91 ± 0,00 | 1,08 ± 0,00 | 103,3 ± 0,1 | 45,2 ± 0,6 |
| NT | | 988,3 ± 0,2 | 13,04 ± 0,15 | 0,70 ± 0,10 | 6,69 ± 0,02 | 3,31 ± 0,00 | 7,21 ± 0,02 | 1,22 ± 0,05 | 1,05 ± 0,00 | 105,2 ± 0,1 | 45,3 ± 0,5 |

## Slide 6
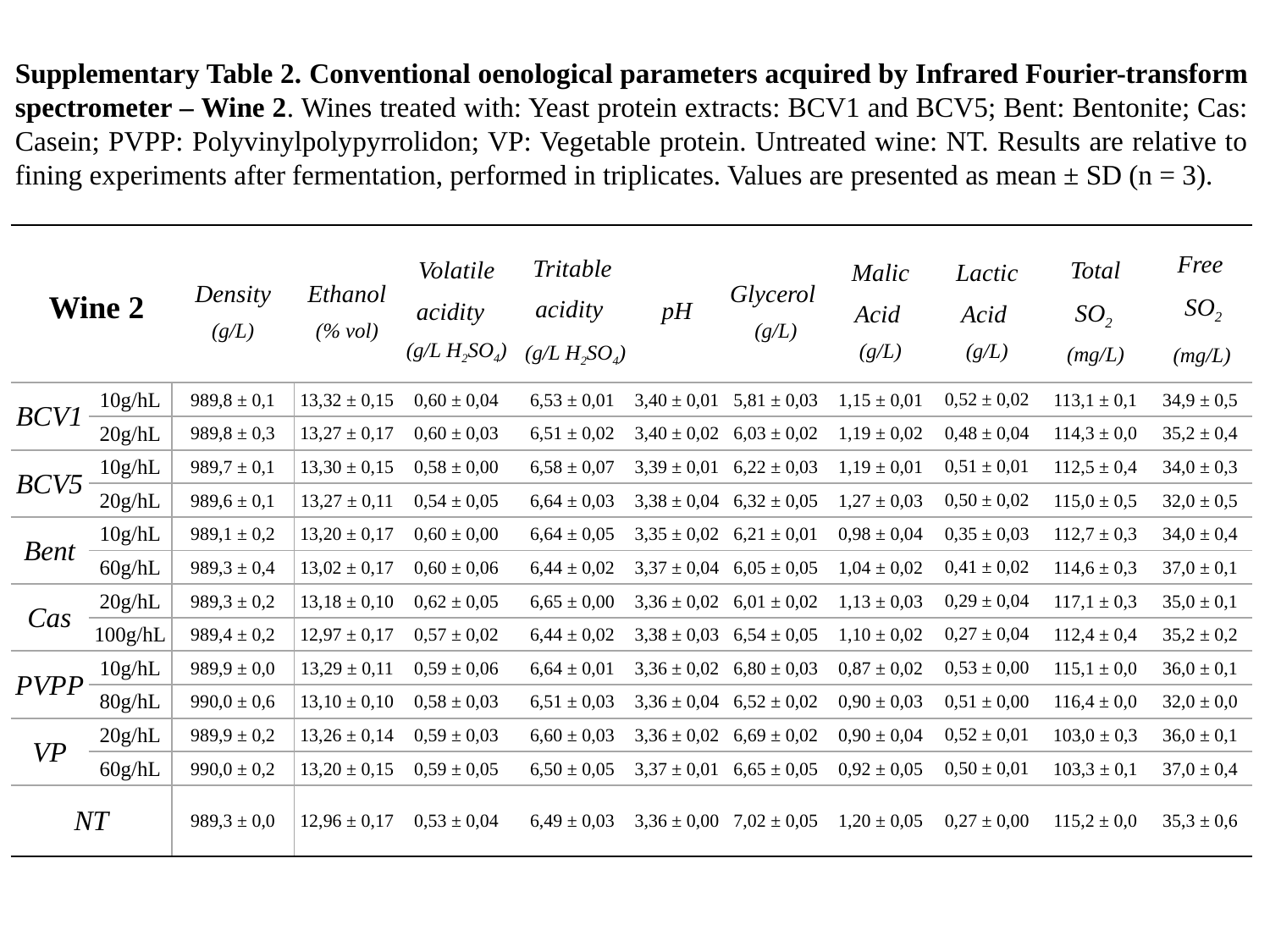

Supplementary Table 2. Conventional oenological parameters acquired by Infrared Fourier-transform spectrometer – Wine 2. Wines treated with: Yeast protein extracts: BCV1 and BCV5; Bent: Bentonite; Cas: Casein; PVPP: Polyvinylpolypyrrolidon; VP: Vegetable protein. Untreated wine: NT. Results are relative to fining experiments after fermentation, performed in triplicates. Values are presented as mean ± SD (n = 3).
| Wine 2 | | Density (g/L) | Ethanol (% vol) | Volatile acidity (g/L H2SO4) | Tritable acidity (g/L H2SO4) | pH | Glycerol (g/L) | Malic Acid (g/L) | Lactic Acid (g/L) | Total SO2 (mg/L) | Free SO2 (mg/L) |
| --- | --- | --- | --- | --- | --- | --- | --- | --- | --- | --- | --- |
| BCV1 | 10g/hL | 989,8 ± 0,1 | 13,32 ± 0,15 | 0,60 ± 0,04 | 6,53 ± 0,01 | 3,40 ± 0,01 | 5,81 ± 0,03 | 1,15 ± 0,01 | 0,52 ± 0,02 | 113,1 ± 0,1 | 34,9 ± 0,5 |
| | 20g/hL | 989,8 ± 0,3 | 13,27 ± 0,17 | 0,60 ± 0,03 | 6,51 ± 0,02 | 3,40 ± 0,02 | 6,03 ± 0,02 | 1,19 ± 0,02 | 0,48 ± 0,04 | 114,3 ± 0,0 | 35,2 ± 0,4 |
| BCV5 | 10g/hL | 989,7 ± 0,1 | 13,30 ± 0,15 | 0,58 ± 0,00 | 6,58 ± 0,07 | 3,39 ± 0,01 | 6,22 ± 0,03 | 1,19 ± 0,01 | 0,51 ± 0,01 | 112,5 ± 0,4 | 34,0 ± 0,3 |
| | 20g/hL | 989,6 ± 0,1 | 13,27 ± 0,11 | 0,54 ± 0,05 | 6,64 ± 0,03 | 3,38 ± 0,04 | 6,32 ± 0,05 | 1,27 ± 0,03 | 0,50 ± 0,02 | 115,0 ± 0,5 | 32,0 ± 0,5 |
| Bent | 10g/hL | 989,1 ± 0,2 | 13,20 ± 0,17 | 0,60 ± 0,00 | 6,64 ± 0,05 | 3,35 ± 0,02 | 6,21 ± 0,01 | 0,98 ± 0,04 | 0,35 ± 0,03 | 112,7 ± 0,3 | 34,0 ± 0,4 |
| | 60g/hL | 989,3 ± 0,4 | 13,02 ± 0,17 | 0,60 ± 0,06 | 6,44 ± 0,02 | 3,37 ± 0,04 | 6,05 ± 0,05 | 1,04 ± 0,02 | 0,41 ± 0,02 | 114,6 ± 0,3 | 37,0 ± 0,1 |
| Cas | 20g/hL | 989,3 ± 0,2 | 13,18 ± 0,10 | 0,62 ± 0,05 | 6,65 ± 0,00 | 3,36 ± 0,02 | 6,01 ± 0,02 | 1,13 ± 0,03 | 0,29 ± 0,04 | 117,1 ± 0,3 | 35,0 ± 0,1 |
| | 100g/hL | 989,4 ± 0,2 | 12,97 ± 0,17 | 0,57 ± 0,02 | 6,44 ± 0,02 | 3,38 ± 0,03 | 6,54 ± 0,05 | 1,10 ± 0,02 | 0,27 ± 0,04 | 112,4 ± 0,4 | 35,2 ± 0,2 |
| PVPP | 10g/hL | 989,9 ± 0,0 | 13,29 ± 0,11 | 0,59 ± 0,06 | 6,64 ± 0,01 | 3,36 ± 0,02 | 6,80 ± 0,03 | 0,87 ± 0,02 | 0,53 ± 0,00 | 115,1 ± 0,0 | 36,0 ± 0,1 |
| | 80g/hL | 990,0 ± 0,6 | 13,10 ± 0,10 | 0,58 ± 0,03 | 6,51 ± 0,03 | 3,36 ± 0,04 | 6,52 ± 0,02 | 0,90 ± 0,03 | 0,51 ± 0,00 | 116,4 ± 0,0 | 32,0 ± 0,0 |
| VP | 20g/hL | 989,9 ± 0,2 | 13,26 ± 0,14 | 0,59 ± 0,03 | 6,60 ± 0,03 | 3,36 ± 0,02 | 6,69 ± 0,02 | 0,90 ± 0,04 | 0,52 ± 0,01 | 103,0 ± 0,3 | 36,0 ± 0,1 |
| | 60g/hL | 990,0 ± 0,2 | 13,20 ± 0,15 | 0,59 ± 0,05 | 6,50 ± 0,05 | 3,37 ± 0,01 | 6,65 ± 0,05 | 0,92 ± 0,05 | 0,50 ± 0,01 | 103,3 ± 0,1 | 37,0 ± 0,4 |
| NT | | 989,3 ± 0,0 | 12,96 ± 0,17 | 0,53 ± 0,04 | 6,49 ± 0,03 | 3,36 ± 0,00 | 7,02 ± 0,05 | 1,20 ± 0,05 | 0,27 ± 0,00 | 115,2 ± 0,0 | 35,3 ± 0,6 |

## Slide 7
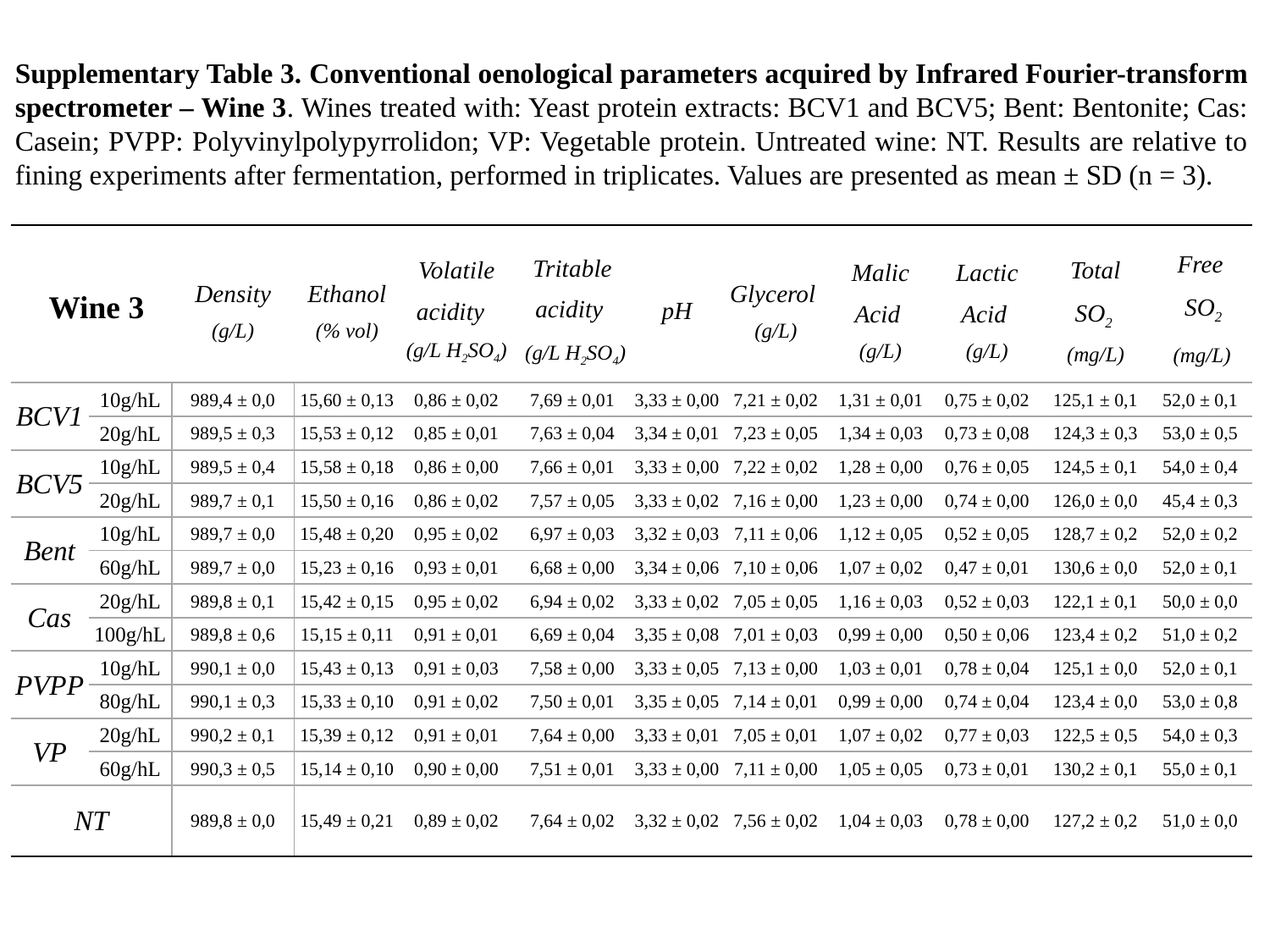

Supplementary Table 3. Conventional oenological parameters acquired by Infrared Fourier-transform spectrometer – Wine 3. Wines treated with: Yeast protein extracts: BCV1 and BCV5; Bent: Bentonite; Cas: Casein; PVPP: Polyvinylpolypyrrolidon; VP: Vegetable protein. Untreated wine: NT. Results are relative to fining experiments after fermentation, performed in triplicates. Values are presented as mean ± SD (n = 3).
| Wine 3 | | Density (g/L) | Ethanol (% vol) | Volatile acidity (g/L H2SO4) | Tritable acidity (g/L H2SO4) | pH | Glycerol (g/L) | Malic Acid (g/L) | Lactic Acid (g/L) | Total SO2 (mg/L) | Free SO2 (mg/L) |
| --- | --- | --- | --- | --- | --- | --- | --- | --- | --- | --- | --- |
| BCV1 | 10g/hL | 989,4 ± 0,0 | 15,60 ± 0,13 | 0,86 ± 0,02 | 7,69 ± 0,01 | 3,33 ± 0,00 | 7,21 ± 0,02 | 1,31 ± 0,01 | 0,75 ± 0,02 | 125,1 ± 0,1 | 52,0 ± 0,1 |
| | 20g/hL | 989,5 ± 0,3 | 15,53 ± 0,12 | 0,85 ± 0,01 | 7,63 ± 0,04 | 3,34 ± 0,01 | 7,23 ± 0,05 | 1,34 ± 0,03 | 0,73 ± 0,08 | 124,3 ± 0,3 | 53,0 ± 0,5 |
| BCV5 | 10g/hL | 989,5 ± 0,4 | 15,58 ± 0,18 | 0,86 ± 0,00 | 7,66 ± 0,01 | 3,33 ± 0,00 | 7,22 ± 0,02 | 1,28 ± 0,00 | 0,76 ± 0,05 | 124,5 ± 0,1 | 54,0 ± 0,4 |
| | 20g/hL | 989,7 ± 0,1 | 15,50 ± 0,16 | 0,86 ± 0,02 | 7,57 ± 0,05 | 3,33 ± 0,02 | 7,16 ± 0,00 | 1,23 ± 0,00 | 0,74 ± 0,00 | 126,0 ± 0,0 | 45,4 ± 0,3 |
| Bent | 10g/hL | 989,7 ± 0,0 | 15,48 ± 0,20 | 0,95 ± 0,02 | 6,97 ± 0,03 | 3,32 ± 0,03 | 7,11 ± 0,06 | 1,12 ± 0,05 | 0,52 ± 0,05 | 128,7 ± 0,2 | 52,0 ± 0,2 |
| | 60g/hL | 989,7 ± 0,0 | 15,23 ± 0,16 | 0,93 ± 0,01 | 6,68 ± 0,00 | 3,34 ± 0,06 | 7,10 ± 0,06 | 1,07 ± 0,02 | 0,47 ± 0,01 | 130,6 ± 0,0 | 52,0 ± 0,1 |
| Cas | 20g/hL | 989,8 ± 0,1 | 15,42 ± 0,15 | 0,95 ± 0,02 | 6,94 ± 0,02 | 3,33 ± 0,02 | 7,05 ± 0,05 | 1,16 ± 0,03 | 0,52 ± 0,03 | 122,1 ± 0,1 | 50,0 ± 0,0 |
| | 100g/hL | 989,8 ± 0,6 | 15,15 ± 0,11 | 0,91 ± 0,01 | 6,69 ± 0,04 | 3,35 ± 0,08 | 7,01 ± 0,03 | 0,99 ± 0,00 | 0,50 ± 0,06 | 123,4 ± 0,2 | 51,0 ± 0,2 |
| PVPP | 10g/hL | 990,1 ± 0,0 | 15,43 ± 0,13 | 0,91 ± 0,03 | 7,58 ± 0,00 | 3,33 ± 0,05 | 7,13 ± 0,00 | 1,03 ± 0,01 | 0,78 ± 0,04 | 125,1 ± 0,0 | 52,0 ± 0,1 |
| | 80g/hL | 990,1 ± 0,3 | 15,33 ± 0,10 | 0,91 ± 0,02 | 7,50 ± 0,01 | 3,35 ± 0,05 | 7,14 ± 0,01 | 0,99 ± 0,00 | 0,74 ± 0,04 | 123,4 ± 0,0 | 53,0 ± 0,8 |
| VP | 20g/hL | 990,2 ± 0,1 | 15,39 ± 0,12 | 0,91 ± 0,01 | 7,64 ± 0,00 | 3,33 ± 0,01 | 7,05 ± 0,01 | 1,07 ± 0,02 | 0,77 ± 0,03 | 122,5 ± 0,5 | 54,0 ± 0,3 |
| | 60g/hL | 990,3 ± 0,5 | 15,14 ± 0,10 | 0,90 ± 0,00 | 7,51 ± 0,01 | 3,33 ± 0,00 | 7,11 ± 0,00 | 1,05 ± 0,05 | 0,73 ± 0,01 | 130,2 ± 0,1 | 55,0 ± 0,1 |
| NT | | 989,8 ± 0,0 | 15,49 ± 0,21 | 0,89 ± 0,02 | 7,64 ± 0,02 | 3,32 ± 0,02 | 7,56 ± 0,02 | 1,04 ± 0,03 | 0,78 ± 0,00 | 127,2 ± 0,2 | 51,0 ± 0,0 |
